# NRT-YOLO: Improved YOLOv5 Based on Nested Residual Transformer for Tiny Remote Sensing Object Detection

**DOI:** 10.3390/s22134953

**Published:** 2022-06-30

**Authors:** Yukuan Liu, Guanglin He, Zehu Wang, Weizhe Li, Hongfei Huang

**Affiliations:** Science and Technology on Electromechanical Dynamic Control Laboratory, Beijing Institute of Technology, Beijing 100081, China; yukuanliu@bit.edu.cn (Y.L.); zehuwang@foxmail.com (Z.W.); 3120210177@bit.edu.cn (W.L.); 3120210170@bit.edu.cn (H.H.)

**Keywords:** tiny object detection, YOLOv5, nested residual transformer, remote sensing imagery

## Abstract

To address the problems of tiny objects and high resolution of object detection in remote sensing imagery, the methods with coarse-grained image cropping have been widely studied. However, these methods are always inefficient and complex due to the two-stage architecture and the huge computation for split images. For these reasons, this article employs YOLO and presents an improved architecture, NRT-YOLO. Specifically, the improvements can be summarized as: extra prediction head and related feature fusion layers; novel nested residual Transformer module, C3NRT; nested residual attention module, C3NRA; and multi-scale testing. The C3NRT module presented in this paper could boost accuracy and reduce complexity of the network at the same time. Moreover, the effectiveness of the proposed method is demonstrated by three kinds of experiments. NRT-YOLO achieves 56.9% mAP_0.5_ with only 38.1 M parameters in the DOTA dataset, exceeding YOLOv5l by 4.5%. Also, the results of different classifications show its excellent ability to detect small sample objects. As for the C3NRT module, the ablation study and comparison experiment verified that it has the largest contribution to accuracy increment (2.7% in mAP_0.5_) among the improvements. In conclusion, NRT-YOLO has excellent performance in accuracy improvement and parameter reduction, which is suitable for tiny remote sensing object detection.

## 1. Introduction

With the introduction of convolutional neural networks (CNNs) [1] into the field of computer vision, object detection techniques have improved significantly. Many excellent algorithms based on CNN were proposed, such as Faster R-CNN [2], YOLO [3] and SSD [4]. Aiming at natural object detection, these methods achieved impressive results in labeled datasets such as MS COCO [5] and PASCAL VOC [6]. In addition, since Transformer components are brought into vision field [7,8], lots of detectors utilizing Transformer, such as Deformable DETR [9] and YOLOS [10], were presented. These methods could provide even higher accuracy than the CNN-based networks in some detection tasks.

Nevertheless, faced with remote sensing images, these general detectors for natural objects always have unsatisfying results due to the features of clustered tiny objects and the high resolution of remote sensing images. Given the high altitude of the imaging sensors, the objects of interest often look tiny and clustered (e.g., there are over 50,000 objects have resolution under 30 × 30 pixels in remote sensing dataset DOTA [11]). Meanwhile, the large size of the input imagery may make detection more difficult. To present more details of the imaging regions, remote sensing images usually have high resolution, such that the maximal resolution can reach 4000 × 4000 pixels in DOTA. As a result, if the detector shrinks the input image to fit the network, the resolution of the tiny objects will be even poorer.

To solve the above problems, image cropping was broadly utilized in remote sensing imagery detection. Many researchers proposed different cropping methods. We generally divide the image cropping methods into four directions:

- Uniform or random cropping [12,13];
- Clustering guided cropping [14,15,16,17];
- Density-map based cropping [18,19,20];
- Reinforcement-learning based cropping [21,22].

The uniform or random cropping methods [12,13] try to address the challenge of ultra high resolution by splitting images in or without order. One can be obviously seen that this kind of cropping approach has less efficiency nor accuracy. Thus, more and more studies began to focus on cropping methods based on clustering [14,15,16,17], density mapping [18,19,20], and reinforcement-learning [21,22]. The detectors utilizing these three manners usually contain two-stage networks: the first are cropping images with coarse-grained detectors which can describe object distribution; and then there is detecting and classifying images with a fine-grained detector. These changes indeed reduce the computing and increase the accuracy compared with the uniform and random methods to some extent.

However, in comparison with the general detectors, the architectures with coarse-grained cropping are still inefficient and complicated. Thus, we attempt to address the tiny object detection problems in remote sensing imagery by utilizing YOLO, the one-stage detector. YOLOv5 has shown its huge potential in detecting tiny objects [23,24,25,26]. By introducing Bi-FPN, [26] it enhances the feature extraction ability of the network. For nightmare remote sensing imagery detection, [25] improves the accuracy using a series of special data augmentation. References [23,24] utilize Transformer components to combine global information with image features, and satisfying test accuracy and speed are achieved.

Inspired by the above works, this study presents a one-stage detection architecture (NRT-YOLO) for tiny remote sensing imagery based on YOLOv5 and Transformer. Specifically, starting from YOLOv5l, we supplement a tiny target prediction head and related feature fusion layers first. A nested residual Transformer (C3NRT) block is then proposed to replace the C3 block in Backbone. It has been verified in this paper that C3NRT could improve accuracy of tiny objects detection while reducing parameters and GFLOPs. Similarly, a nested residual attention (C3NRA) block is presented and utilized before the detection layer. We also implement the multi-scale testing method in our training. Consequently, compared with YOLOv5l, NRT-YOLO improves mAP_0.5_ by 4.5% with image size of 1024 × 1024 pixels in DOTA dataset, reaching 56.9%. Furthermore, the proposed architecture has less parameters (38.1 M) and almost the same GFLOPs (115.2). The main contributions of this paper can be concluded as follows:

1. A one-stage detector is proposed to boost efficiency and reduce complexity for remote sensing object detection;
2. A nested residual Transformer module is proposed to acquire global information, and it is verified that the structure boosts the accuracy of tiny object detection and reduces complexity;
3. Some efficient improvements and tricks are added in YOLOv5 to address tiny object problems, such as an extra prediction head, nested residual attention and multi-scale testing.

The rest of the paper is organized as follows: Section 2 introduces the related work including YOLOv5, Transformer and the attention mechanism; Section 3 presents the NRT-YOLO architecture, the improved YOLOv5 network for remote sensing imagery and the detailed components; and Section 4 gives three experiment examples to demonstrate the accuracy and efficiency of the proposed algorithm.

## 2. Related Work

In order to facilitate the later design of the object detection architecture for remote sensing imagery, the related work including YOLOv5, Transformer and the attention mechanism are introduced in this section.

### 2.1. YOLOv5

The YOLO family [3,27,28,29] is a famous object detection method based on a one-stage network. YOLOv5 is the latest architecture in the series, which combines the structures of SPPF, the Path Aggregation Network (PANet), the BottleNeck and residual network, etc. The largest model of YOLOv5 reaches 68.9% mAP on the MS COCO dataset with the speed of 12.1 ms in NVIDIA V100 b1 GPU.

**Figure 1:**
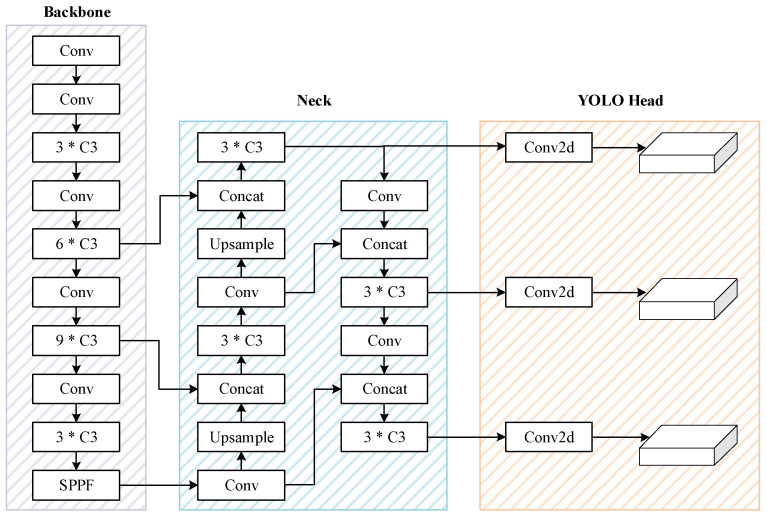
The architecture of YOLOv5 v6.1.

**Figure 2:**
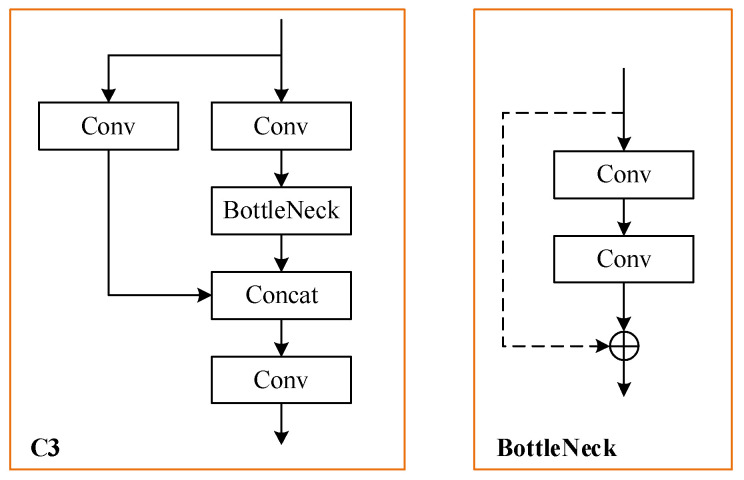
The structures of C3 and BottleNeck.

There are five types of YOLOv5 with different width and depths of network: YOLOv5n, YOLOv5s, YOLOv5m, YOLOv5l and YOLOv5x. Among these models, YOLOv5l is the basic network, and has both excellent accuracy and high speed in object detection. Therefore, YOLOv5l is used as the baseline network in this paper. The architecture of YOLOv5 v6.1 is illustrated in Figure 1. Furthermore, the detailed structures of C3 and BottleNeck modules are shown in Figure 2.

There are two primary drawbacks in the YOLOv5 architecture when it is used to detect tiny objects:

1. It lacks shallow network information as a consequence of the neglect of features in the first C3 block;
2. It lacks the ability to obtain global and contextual information which can benefit the network with regard to accuracy and efficiency.

### 2.2. Vision Transformer

Vision Transformer (ViT) [7] is the first successful attempt to introduce Transformer [30] into the computer vision field. A ViT architecture mainly consists of the following modules: positional encoding; multi-head attention; feed forward, and other necessary components like normalization, dropout and residual layers.

The process of imaging classification of ViT can be summarized as follows: first, the image is reshaped into a sequence of flattened patches; also, extra positional information is added using a positional encoding module; and then the sequence with the learnable position encoding is inputted to Transformer encoders for computing global attention and extracting features by utilizing the multi-head attention module; finally, the predicted class is given through an MLP head layer.

ViT verifies that pure Transformer architecture can also achieve excellent results in computer vision tasks. It even has a better performance than CNNs when training with larger data volumes. However, there are two obvious disadvantages:

1. It requires ultra-scale datasets to reach its maximum potential without the capability of inductive bias of CNNs;
2. If the input image size becomes larger, the sequence will become longer, which may significantly increase complexity as a consequence.

### 2.3. Attention Mechanism

Currently, attention mechanism has been widely applied in many fields of deep learning. By making relevant and irrelevant choices of information features, the module can build dynamic weights, which helps the network to record the location relationships and estimate the importance of different information. As a result, the useless information is weakened while the important information is strengthened. The efficiency of the network can then be improved.

Attention mechanisms can be basically divided into four types [31], and two of them are broadly utilized in object detection:

- Channel attention, which focuses on important objects, such as SENet [32];
- Spatial attention, which focuses on important locations, such as self-attention in Transformer and DCN [33];

The channel attention mechanism aims to extract the useful information of the feature map, and the spatial attention mechanism focuses more on the important location information. Also, there exists some mixed attention mechanisms such as CBAM [34]. CBAM is a hybrid attention module that combines channel attention and spatial attention. The mixture of the two types helps CBAM perform well in improving model accuracy and suppressing irrelevant noise information.

## 3. Approach

To address the problems of networks based on YOLOv5 and ViT in detecting tiny objects, we propose an improved YOLO algorithm (NRT-YOLO) utilizing a nested residual Transformer in this section. The overall architecture of NRT-YOLO is given first. Furthermore, the important components of NRT-YOLO are demonstrated, including a tiny object prediction head, a nested residual Transformer module and a nested residual attention module.

### 3.1. Overview of NRT-YOLO

**Figure 3:**
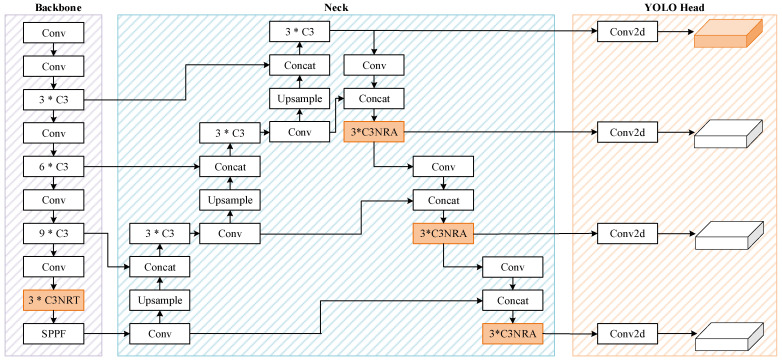
The architecture of the proposed NRT-YOLO.

The architecture of the proposed method is illustrated in Figure 3. The general goal of the network established is to address the two problems of YOLOv5. For the problem that YOLOv5 lacks shallow network information, we add a tiny prediction head and related feature fusion layers. And for the problem that it lacks the capability to acquire global and contextual information, a nested residual Transformer module named C3NRT and a similar attention block are proposed. Meanwhile, the combination with CNNs could avoid the disadvantages of complexity and mass-data driven of ViT.

### 3.2. Tiny Object Prediction Head

An extra object prediction head is added to the YOLO head and its related feature fusion layers is added in the Neck network.

Obviously, the added head and related layers can fuse the shallow information of the Backbone network. Throughout the downsampling process with stride 2 in the Backbone, the network can acquire more semantic information, but loses a large amount of detailed feature information. However, the detailed information contains mass features of tiny size objects, and that may be ignored in the downsampling process.

Therefore, utilizing a skip connection, an additional feature fusion structure is added to the Neck to fuse shallow layers in Backbone which have larger feature maps and contain more detailed information. And a new object prediction head is introduced based on this structure to enhance the network’s ability to detect tiny objects. Finally, we get a four-head predictor structure which could detect both tiny objects and multi-scale objects. Although the extra prediction head will increase the complexity of the network in parameters and GFLOPs, it can greatly improve the detection performance for tiny objects.

### 3.3. Nested Residual Transformer (C3NRT) Module

**Figure 4:**
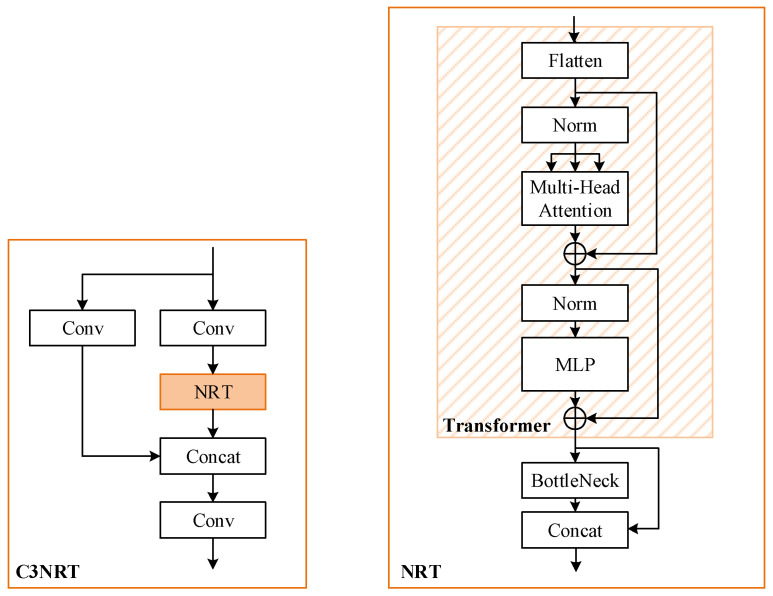
The structures of C3NRT and NRT.

To improve YOLOv5 in global information acquisition, the Transformer block is introduced. And inspired by reference [23,35], we propose a nested residual network structure combining Transformer and BottleNeck called C3NRT. The proposed module can not only improve the performance of the Transformer block in CNNs but also reduce parameters and GFLOPs. Specifically, the structure of C3NRT is shown in Figure 4.

#### 3.3.1. Transformer Encoder Block

Combing CNN with ViT can improve the ability of global information sensing of CNN and reduce the complexity of ViT. In this paper, a classical Transformer encoder is used, and the details can be described as follows.

1. 1. Flatten

The feature map should be flattened to sequence first for following operations such as linear mapping.

2. Multi-head attention

Multi-Head attention is the most important layer of the Transformer encoder structure. The feature map sequence is normalized by norm layer and then passed to the multi-head attention layer which is composed of single-head self-attention.

Utilizing three-times linear mapping, the single-head self-attention mechanism contains three fully connected layers, which are used as query (*Q*) matrix, key (*K*) matrix and value (*V*) matrix, respectively. After matrix multiplication, scale, mask and softmax operations, the weights of sequences *Q* and *K* are outputted to multiply with *V*. Thus, the sequence with attention is produced. The attention weights determine which parts of its sequence the model should focus on, and can effectively improve the detection efficiency and accuracy. The output result of single-head self-attention can be expressed by:

(1) $$\mathrm{Attention}\left(Q,\;K,\;V\right)=\mathrm{softmax}\left(\frac{QK^{T}}{\sqrt{d_{K}}}\right)V$$

where $d_{K}$ is the dimension of K.

After contact layer and linear mapping, multiple single-head self-attention mechanisms can be combined into a multi-head attention block. Each head of the multi-head attention has different *Q*, *K* and *V*, which can initialize and weight randomly and separately. This process enables the entire attention block to synthesize different self-attention information in different relational subspaces. The output of the multi-head attention layer can be described as:

(2) $$\mathrm{MultiHead}\left(Q,\;K,\;V\right)=\mathrm{Concat}({\mathrm{head}}_{1},\;\ldots ,{\;\mathrm{head}}_{h})W^{O}\;{\;\;\;\mathrm{where}\;\mathrm{head}}_{i}=\mathrm{Attention}\left(QW_{i}{}^{Q},\;KW_{i}{}^{K},\;VW_{i}{}^{V}\right)$$

where $W^{O}$, $W_{i}{}^{Q}$, $W_{i}{}^{K}$, and $W_{i}{}^{V}$ are weight matrixes; and satisfy $W_{i}{}^{Q}\in {\mathbb{R}}^{d_{\mathrm{model}}\times d_{K}}$, $W_{i}{}^{K}\in {\mathbb{R}}^{d_{\mathrm{model}}\times d_{K}}$, $W_{i}{}^{V}\in {\mathbb{R}}^{d_{\mathrm{model}}\times d_{V}}$, and $W^{O}\in {\mathbb{R}}^{hd_{K}\times d_{\mathrm{model}}}$, respectively; *h* is the head number of multi-head attention layer; $d_{\mathrm{model}}$ and $d_{V}$ are the dimensions of model and V, respectively. In this paper, h is set as 4.

3. MLP

The input of the multilayer perceptron (MLP) comes from the output of the normalized multi-headed attention mechanism. MLP consists of two fully connected layers and the ReLu activation function. The MLP process can be formulated as:

(3) $$\mathrm{MLP}\left(x\right)=\mathrm{ReLu}(xW_{1}+b_{1})W_{2}+b_{2}$$

where *x* is the input sequence; $W_{1}$, $W_{2}$, and $b_{1}$, $b_{2}$, are weights and biases of the two fully connected layers, respectively.

#### 3.3.2. C3NRT Module

**Figure 5:**
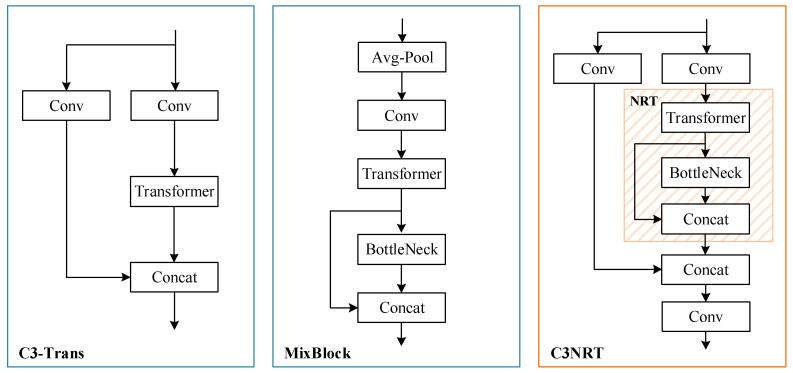
The comparation between C3NRT and other structures.

To combine Transformer with CNNs, several different manners are suggested in previous works. Reference [23] presents C3-Trans, which replaces original BottleNeck block in C3 by Transformer block. And a MixBlock structure of Transformer and BottleNeck is studied in [35], which has proved to be more efficient. Inspired by them, we propose C3NRT, considering both high efficiency and low complexity. The structures of the above three modules are demonstrated in Figure 5.

As illustrated in Figure 5, the combined structure of Transformer and BottleNeck is utilized to substitute for BottleNeck in the proposed C3NRT. The details are described as follows:

1. Nested residual architecture

Essentially, the C3NRT module is a nesting of multiple residual structures [36]. C3NRT, NRT, and BottleNeck modules all have the forms of the residual net. The residual architecture can improve efficiency, reduce parameters and avoid some gradient problems.

For traditional convolutional neural networks, as the network becomes deeper and deeper, it often brings the problems of gradient disappearance and gradient explosion. These phenomena will make the optimization of the network become more and more difficult. Although batch normalization can solve this problem to some extent, it degrades the performance of the network. That means the training error will become larger and larger with the increase in network depth and eventually lead to huge deviations.

The residual structure is proposed to solve the above problems. Utilizing skip connection, residual networks can address the gradient and degradation problems when deepening the network.

In the C3NRT module, the outermost residual structure is similar to C3, consisting of three convolutional layers and an NRT block. The output of NRT is concatenated with the shallow network information and then passed through a convolutional layer. The middle residual structure is the NRT module which consists of a Transformer encoder and a BottleNeck module. In this structure, passed through Transformer and BottleNeck, the feature map is concatenated with the previous feature map. The innermost residual structure is a BottleNeck network. It contains two convolutional layers and an Add operation, which can fuse the shallow and deep features and maintain the numbers of features and channels after fusion.

2. Module location

A C3NRT module is utilized at the end of YOLOv5 Backbone in our study. The Backbone network locates on a shallow level among the entire architecture. Thus, the Transformer block could improve the network’s ability to obtain global information with maximum efficiency. Furthermore, some detailed feature information may be lost in the feature fusion process of SPPF, so it will be better to assign attention weights adaptively before the SPPF module. However, the testing of Transformer requires a large amount of memory. To save the expensive training resources and reduce costs, this paper only utilizes the nested residual Transformer module at the position where it has the lowest resolution feature map in Backbone.

3. Size of feature map

The input and output feature maps of C3NRT have the same sizes. Also, the resolution of the feature map is maintained throughout the forward process. When the input image size is 1024 × 1024 × 3 (width × height × channel), the input and output feature map sizes of C3NRT module are 32 × 32 × 1024. And the input sizes of the Transformer encoder block and the BottleNeck module are 32 × 32 × 512 and 32 × 32 × 256, respectively.

As for the MixBlock module, it seems to have larger sizes. Assuming its average pooling stride is 1, and the convolutional kernel size is 1 × 1 and the channel is 1024, its output can reach 32 × 32 × 1024. At this time, the sizes of the feature map input to Transformer and BottleNeck modules are 32 × 32 × 1024 and 32 × 32 × 512, respectively. Compared with that in C3NRT, the sizes increase greatly. That means C3NRT will have less parameters and GFLOPs than the MixBlock.

In conclusion, compared with the other two structures in Figure 5, the proposed C3NRT could improve the efficiency of Transformer block and reduce the complexity at the same time.

### 3.4. Nested Residual Attention

**Figure 6:**
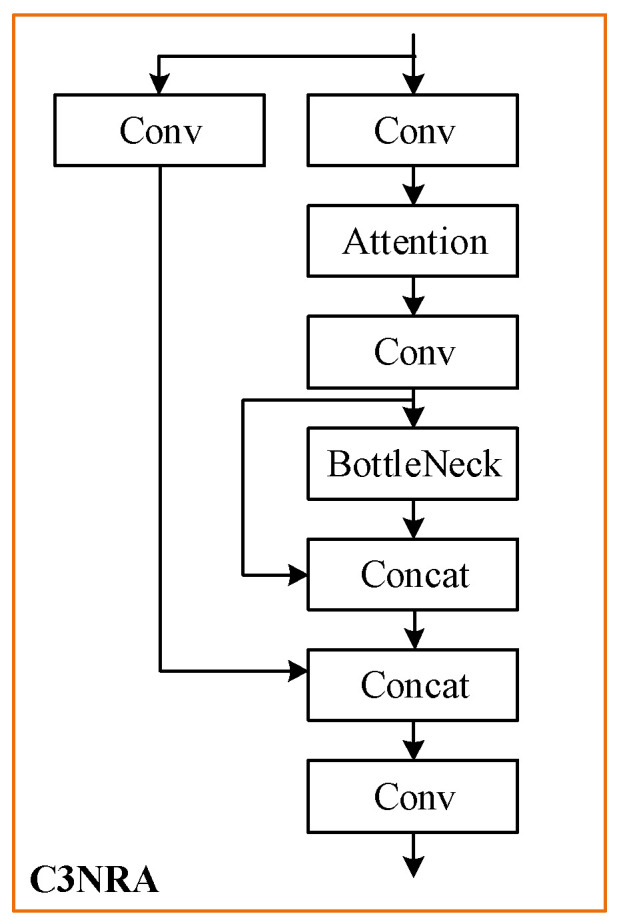
The structure of C3NRA.

Similarly, based on the proposed nested residual Transformer module, a nested residual attention mechanism (C3NRA) is presented, as shown in Figure 6.

Similar to C3NRT, C3NRA is able to enhance the performance of the attention block further and reduce the complexity at the same time.

We employ CBAM [34] in the designed attention block. The process of CBAM can be summarized as follows: for an input feature map, CBAM first generates the channel attention feature through its average and maximum pooling, and transfers the feature map to sequence and passes it to the MLP layer, and then processes it in the spatial dimension to obtain spatial information; finally, multiplies the previous feature map by the output to modify the attention weights adaptively.

### 3.5. Multi-Scale Testing

Multi-scale testing is a trick which can improve the detection model significantly. By changing the size of the input image randomly, the model could have better accuracy and robustness. Large size imagery has contribution to accuracy due to its more information about tiny objects; and small size imagery has contribution to applicability scale for images with different resolutions. The scale of the multi-scale testing is set as $\left[0.5,\;1.5\right]$ in this paper.

## 4. Experiments

In this section, the DOTA dataset utilized in this paper is introduced. Also, the implementation details and evaluation metrics are declared. Thus, to verify the performance of the proposed method, three experiments are demonstrated: comparison between NRT-YOLO and other algorithms; ablation study; and comparison between C3NRT and other transformer blocks. Finally, the excellent properties of precision and model size are concluded from the experiments.

### 4.1. Dataset

**Figure 7:**
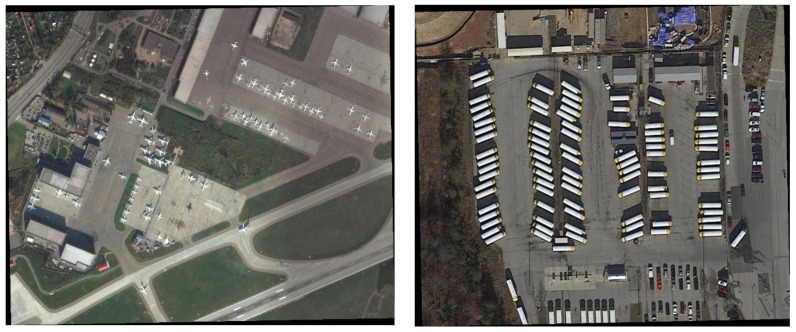
Sample images in DOTA.

In order to verify the performance of the proposed algorithm in detecting tiny remote sensing objects, the DOTA v1.5 [11] dataset is utilized in this paper. DOTA v1.5 is a large-scale dataset with 2806 remote sensing pictures. Also, the image sizes of the dataset are large, from 800 × 800 to 4000 × 4000 pixels. Figure 7 shows some samples of DOTA.

**Figure 8:**
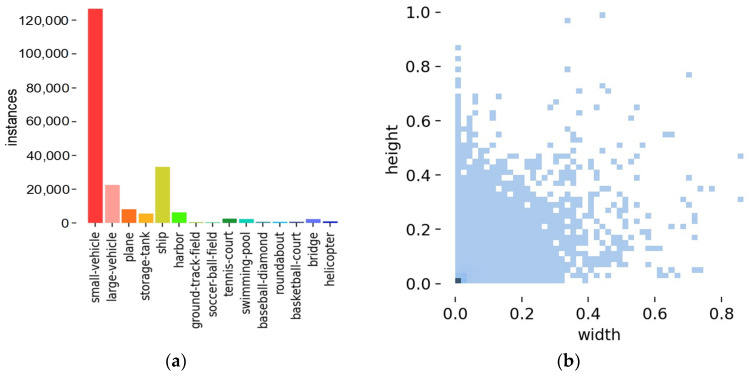
Distribution of classes and sizes of objects in DOTAv1.5: (**a**) Distribution histogram of the classes of the labels; (**b**) Heat map of the size of the objects.

DOTA v1.5 contains 188,282 objects and 15 classes such as small-vehicle, plane, helicopter, etc. The distribution of classifications and the size of objects of the training set are shown in Figure 8. It can be concluded from Figure 8 that DOTA v1.5 is a challenging dataset with unbalanced samples and vast tiny objects. From Figure 8a, the class small-vehicle has over 120,000 objects. Contrarily, some classes such as helicopter and basketball-court have no more than 1000 objects. In addition, it is shown in Figure 8b that the sizes of objects in DOTA are barely over 5% of the image size.

### 4.2. Implementation Details and Evaluation Metrics

1. Implementation details

Before testing our models, we pre-trained YOLOv5l on the DOTA dataset to utilize the weights. Based on the pre-trained weights, we can shrink our training epochs to 80. In addition, the training batch size is two, and the input image size is 1024 × 1024 pixels. As with the hyperparameter settings of YOLOv5l, we employ an SGD optimizer with 0.937 in momentum and 0.0005 in weight decay. As for the learning rate, it is set as 0.001 in the first three warmup epochs, then achieves 0.01 and shrinks to 0.0001 continuously until the final cycle.

In addition, several data augmentation strategies are utilized in the implementation, including: Moasic; random affine with 0.5 of scale ratio and 0.1 translation ratio; augment HSV; and random horizontal flip with 50% probability.

All of the tests in this paper are finished based on NVIDIA RTX3080 GPU.

2. Evaluation metrics.

During the experiments in this paper, ${\mathrm{mAP}}_{0.5}$ and mAP are the metrics that we pay most attention to. These property evaluation metrics for model testing can be described as:

(4) $${\mathrm{mAP}}_{0.5}=\frac{1}{n_{class}}\int_{0}^{1}P\left(R\right)dR$$

(5) $$\mathrm{mAP}=\mathrm{avg}\left({\mathrm{mAP}}_{i}\right),\;i=0.5:0.05:0.95$$

where $n_{class}$ represent the number of the classes; *P* and *R* represent precision and recall, respectively, and they satisfy:

(6) $$P=\frac{TP}{TP+FP}$$

(7) $$R=\frac{TP}{TP+FN}$$

where *TP* represents the number of the prediction boxes whose IoU > 0.5; *FP* represents the number of the prediction boxes whose IoU ≤ 0.5; and *FN* represents the number of the labels without prediction.

In addition, parameters (the total number of weight parameters among all layers) and GFLOPs are used to evaluate the complexity of the network.

### 4.3. Experiment Results

**Figure 9:**
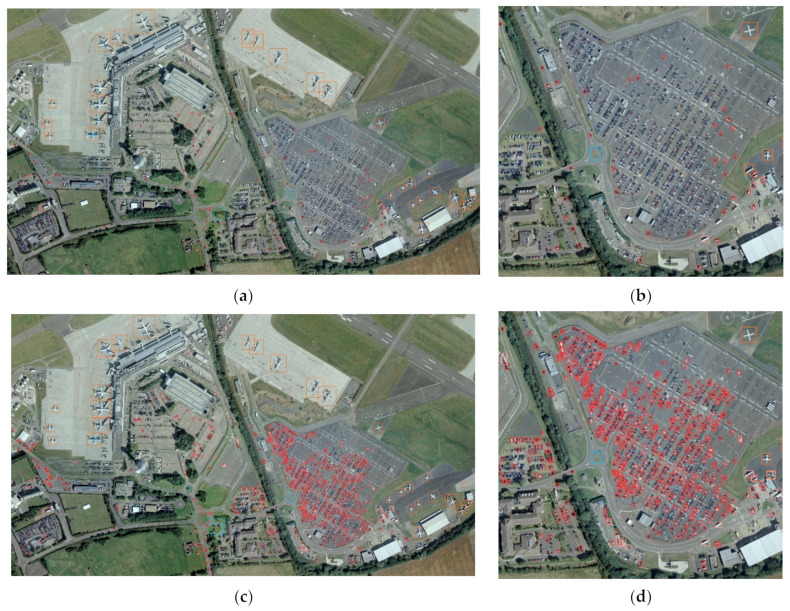
The detection examples: (**a**) Detection result of an example from DOTA validation set using YOLOv5l; (**b**) Detail view of (**a**); (**c**) Detection of the same picture; (**d**) Detail view of (**c**).

Using the implement setup given in Section 4.2, we evaluate NRT-YOLO in terms of precision, recall, mAP_0.5_, mAP, parameters, GFLOPs and latency (inference time). Furthermore, to reflect the good properties of the proposed method, some methods from the YOLO family are used to contrast, including YOLOv4, YOLOv5m, YOLOv5l and YOLOv5x. The general comparison results are listed in Table 1. The results of the COCO evaluation [5] are listed in Table 2. The mAP_0.5_ comparison results of different classifications are listed in Table 3. And the detection examples of the DOTA validation set is illustrated in Figure 9.

Table 1 details the results of different metrics of the five object detection algorithms. Overall, NRT-YOLO could achieve the highest mAP_0.5_ and mAP with only half parameters and GFLOPs of YOLOv5x. The mAP_0.5_ and mAP of NRT-YOLO in DOTA are 56.9% and 33.2%, respectively, 2.8% and 1.0% higher than YOLOv5x, and 4.5% and 1.8% higher than YOLOv5l, respectively. Moreover, the recall of the proposed method reaches 51.5%, which is much higher than YOLOv5l (47.6%). This indicates that the improved architecture could greatly reduce the missed detection rate, which means better performance in tiny object detection. As for the model complexity, the parameters of NRT-YOLO are only 17.2 million (M) larger than the smallest one, and 46% smaller than YOLOv5x. The GFLOPs of YOLOv5l and NRT-YOLO are nearly the same, which means the good property of computing of NRT-YOLO. In addition, though NRT-YOLO increases the inference time by 6.8 ms, reaching 48.7 ms, it is still smaller than that of YOLOv5x (51.6 ms).

In COCO evaluation metrics, mAP^small^, mAP^medium^, and mAP^large^ stand for the mAP of objects with sizes of (0, 32^2^], (32^2^, 96^2^] and (96^2^, ꝏ) pixels, respectively. From Table 2, we can see that NRT-YOLO has the best results in all three metrics among the listed methods. In small object detection, in comparison of YOLOv5l, NRT-YOLO improves mAP by 4.0%, reaching 13.0 and thereby exceeding YOLOv5x by 2.4%. It illustrates the ability of the proposed architecture in detecting tiny objects. As for medium and large objects, NRT-YOLO also performs well: it increases mAP by 2.6% and 4.0% respectively compared with the baseline.

Table 3 lists the results of mAP_0.5_ of the five methods under each DOTA object classification. It can be concluded that NRT-YOLO has the best detection performance in most classifications, especially tiny object classifications such as a small-vehicle. As for the objects with large scale variations like aircraft, ships and large vehicles, NRT-YOLO also has better detection performance. In addition, the proposed method has an excellent performance for the small sample objects: its mAP_0.5_ achieves 27.7%, which is five times higher than the second best method. Based on the additional Transformer module and attention mechanism module, the proposed method has a much higher property in small sample object detection.

Figure 9 represents the comparison of detection results of YOLOv5l and NRT-YOLO for a DOTA validation image. Figure 9a,b demonstrate the detection result of YOLOv5l and its detail view, respectively. Figure 9c,d show the detection result of NRT-YOLO and its detail view. The red prediction boxes stand for a small-vehicle whose sizes are smaller than 50 pixels. It can be obviously seen from Figure 9b,d that NRT-YOLO has a much better result in tiny object detection.

### 4.4. Ablation Study

There are many improvement measures in NRT-YOLO, including: adding a tiny prediction head. replacing C3 with a C3NRT module, introducing C3NRA, and multi-scale testing. To verify the effect of these measures on NRT-YOLO, an ablation experiment is undertaken in this paper. The results of the ablation study are listed in Table 3 and Table 4.

As shown in Table 4, the added tiny prediction head has 1 M parameters, about 20 GFLOPs and 6 ms increments, but also 1% increment in mAP_0.5_. In addition, the nested residual Transformer module proposed in this paper contributes largely in improving the network performance. The mAP_0.5_ and mAP_metrics_ are boosted by 2.7% and 1.9%, respectively. C3NRT increases inference time to 49.9 ms due to the linear mapping process introduced by the Transformer architecture. However, the Transformer module also minimizes the complexity of the network: the 2.7 M parameters and 1.8 GFLOPs are reduced. The parameters and GFLOPs are reduced further by utilizing C3NRA: 6.4 M and 12.2, respectively. The C3NRA module also cuts down the inference time by 1.2 ms. The parameters shrink by 21% in comparison with the base algorithm utilizing C3NRT and C3NRA, and the GFLOPs are nearly the same with YOLOv5l. The multi-scale testing brought 0.9% and 0.1% improvement of mAP_0.5_ and mAP, respectively.

Table 5 presents the impact of each measure on mAP_0.5_ for all classifications. It can be observed that C3NRT has a determined contribution to the entire network. This module contributes a more than 1% mAP_0.5_ increment in 11 classifications. In particular, the improvement reaches 19.5% in the helicopter class which only has small samples. As for ground track field and baseball diamond, the classifications which often accompanied with complex background, mAP_0.5_ are improved by 3.5% and 2.8%. Furthermore, the extra prediction head helps the network detect small vehicles with a 1.1% improvement mAP_0.5_. In addition, C3NRA raises mAP_0.5_ by 3.5% for helicopter detection.

### 4.5. Comparison between NRT and Other Transformer Blocks

To further contrast the three Transformer blocks demonstrated in Figure 5, a comparison experiment is conducted. In this experiment, the YOLOv5l with extra prediction head is set as the baseline. On this basis, we replace the last C3 block of Backbone with these three Transformer blocks. The other conditions are as same as Section 4.2. The comparison results between C3NRT and the other two Transformer blocks are listed in Table 6.

From Table 6, it can be observed that the C3NRT module achieves nearly the highest detection accuracy with the smallest parameters. As mixed structures of Transformer and BottleNeck, MixBlock and C3NRT have excellent performance in accuracy, and both of them reach 56.1% mAP_0.5_. Compared with C3-Trans, there is a huge increment (2.5%). Furthermore, in comparison of C3-Trans, C3NRT improved 2.3%, 1.1% and 1.8% in precision, recall and mAP, respectively. As for the complexity metrics, C3NRT has much less parameters than C3-Trans (46.7 M) and MixBlock (59 M), reaching 44.5 M. This is because the feature map sizes of the Transformer encoder in these three blocks are different. The input feature map sizes of the Transformer encoder are 32 × 32 × 512, 32 × 32 × 512 and 32 × 32 × 1024, respectively. And the output sizes are 32 × 32 × 256, 32 × 32 × 512 and 32 × 32 × 512. In addition, the GFLOPs of C3-Trans and C3NRT are nearly the same, at 124.9 and 126.0, respectively.

## 5. Conclusions

To reduce the complexity and improve the efficiency of the two-stage detectors for tiny remote sensing object detection, this paper presents an improved YOLOv5 method based on a nested residual Transformer. NRT-YOLO, the proposed method, is much simpler in comparison with the detectors with image cropping networks for remote sensing imagery. The features of NRT-YOLO can be summarized as an extra prediction head for tiny objects; a novel nested residual Transformer module, C3NRT; a nested residual attention module, C3NRA; and multi-scale testing.

Furthermore, to verify the effectiveness of those improvements, three kinds of experiments were conducted on the DOTA dataset with a 1024 × 1024 sized image. Compared with YOLOv5l, NRT-YOLO increases mAP_0.5_ by 4.5%, reaching 56.9%; it reduces parameters by 8.1 M to 38.1 M; and it nearly remains the same with regard to GFLOPs, at 115.2. NRT-YOLO shows excellent performance in small sample object detection, and the mAP_0.5_ of helicopter classification in the DOTA dataset of the proposed method is improved by five times, achieving 27.7%. In ablation study, the C3NRT module was demonstrated to contribute the largest accuracy increment (2.7% in mAP_0.5_, and 1.9% in mAP) among the improvements. It can be also concluded that C3NRT could significantly reduce the parameters and GFLOPs of the network. As for the comparison experiment for Transformer blocks, the proposed module C3NRT is verified to have excellent performance in both accuracy improvement and low complexity.

NRT-YOLO is suitable for tiny remote sensing objects because of its high accuracy and low complexity. In future studies, more different datasets will be employed to testify the generalization capability of the NRT-YOLO and C3NRT modules.

## Figures and Tables

**Table 1:** Comparison results between NRT-YOLO and other YOLO methods.

Method	P (%)	R (%)	mAP_0.5_ (%)	mAP (%)	Parameters (M)	GFLOPs	Latency (ms)
YOLOv4	76.8	43.2	48.1	27.6	26.9	51.0	37.8
YOLOv5m	79.0	46.2	51.0	30.2	20.9 *	**48.1**	**35.2**
YOLOv5l	79.3	47.6	52.4	31.4	46.2	108.0	41.9
YOLOv5x	**81.5**	49.2	54.1	32.2	86.3	204.3	51.6
NRT-YOLO	78.1	**51.5**	**56.9**	**33.2**	38.1	115.2	48.7

* The best results of every metric are bolded.

**Table 2:** Comparison results of COCO evaluation.

Method	mAP^small^ (%)	mAP^medium^ (%)	mAP^large^ (%)
YOLOv4	6.7	21.5	39.1
YOLOv5m	8.3	23.9	42.5
YOLOv5l	9.0	25.8	43.2
YOLOv5x	10.6	26.8	46.6
NRT-YOLO	13.0 *	**28.4**	**47.2**

* The best results of every metric are bolded.

**Table 3:** Comparison results (mAP_0.5_) of different classifications between NRT-YOLO and other YOLO methods.

Class	YOLOv4	YOLOv5m	YOLOv5l	YOLOv5x	NRT-YOLO
Small vehicle	28.6	31.6	31.9	32.1	33.2 *
Large vehicle	66.0	69.0	68.8	68.7	**71.7**
Plane	77.2	79.9	80.0	79.3	**82.6**
Storage tank	42.4	50.2	44.6	46.4	**52.2**
Ship	56.9	58.8	61.0	61.5	**65.6**
Harbor	69.3	71.2	72.0	**76.3**	72.4
Ground track field	32.8	35.9	41.8	40.3	**48.8**
Soccer ball field	38.2	38.2	38.6	**43.5**	38.1
Tennis court	90.4	93.0	92.8	**93.9**	93.0
Swimming pool	49.9	53.7	54.7	57.1	**57.2**
Baseball diamond	59.1	61.4	68.2	69.9	**71.8**
Roundabout	36.7	37.0	42.2	**44.1**	43.3
Basketball-court	45.2	47.8	49.3	51.0	**55.5**
Bridge	27.4	34.9	35.6	**41.6**	39.9
Helicopter	1.5	2.5	5.0	5.6	**27.7**

* The best results of each classification are bolded.

**Table 4:** Results of ablation study.

Method	mAP_0.5_ (%)	mAP (%)	Parameters (M)	GFLOPs	Latency (ms)
YOLOv5l-1024	52.4	31.4	46.2	108.0	41.9
+prediction head	53.4 (+1.0) *	31.5 (+0.1)	47.2	127.8	47.9
+C3NRT	56.1 (+2.7)	33.4 (+1.9)	44.5	126.0	49.9
+C3NRA	56.0 (−0.1)	33.4 (−−)	37.9	113.8	48.7
+ms testing	56.9 (+0.9)	33.5 (+0.1)	37.9	113.8	48.7

* The increments of mAP_0.5_ and mAP are noted in parentheses.

**Table 5:** Ablation study of different classifications (mAP_0.5_).

Class	YOLOv5l	+Detection Head	+C3NRT	+C3NRA	+ms Testing
Small vehicle	31.9	33.0 *	33.4	33.3	33.2
Large vehicle	68.8	**71.4**	71.7	70.9	71.7
Plane	80.0	80.6	80.8	81.2	**82.6**
Storage tank	44.6	**51.0**	**52.9**	53.7	52.2
Ship	61.0	**65.4**	**66.5**	66.3	65.6
Harbor	72.0	**75.6**	74.6	74.6	72.4
Ground track field	41.8	42.0	**45.5**	44.7	**48.8**
Soccer ball field	38.6	36.0	**38.1**	37.6	38.1
Tennis court	92.8	91.7	**92.8**	92.6	93.0
Swimming pool	54.7	54.4	**56.1**	54.7	**57.2**
Baseball diamond	68.2	67.8	**70.6**	68.9	**71.8**
Roundabout	42.2	37.9	**38.9**	40.5	**43.3**
Basketball-court	49.3	**50.4**	**52.4**	52.7	**55.5**
Bridge	35.6	**37.5**	**41.2**	39.7	39.9
Helicopter	5.0	6.2	**25.7**	**29.2**	27.7

* The data with improvement over 1% compared to previous measure is bolded.

**Table 6:** Comparison results between C3NRT and other Transformer blocks.

Method	P (%)	R (%)	mAP_0.5_ (%)	mAP (%)	Parameters (M)	GFLOPs
C3-Trans [23]	78.8	49.1	53.5	31.6	46.7	124.9 *
MixBlock [35]	80.1	**50.5**	56.1	**33.5**	59.0	138.7
C3NRT	**81.1**	50.2	**56.1**	33.4	**44.5**	126.0

* The best results of every metric are bolded.

## Data Availability

The data presented in this study are available on request from the corresponding author.
